# One Health Gathering: Arctic Indigenous Peoples Voices and Perspectives

**DOI:** 10.1186/s12919-025-00329-1

**Published:** 2025-06-16

**Authors:** G. K. Healey Akearok, C. V. L. Larsen

**Affiliations:** 1 Qaujigiartiit Health Research Centre, Iqaluit, NU Canada; 2 Centre for Public Health in Greenland, National Institute of Public Health, University of Southern Denmark, Copenhagen, Denmark; 3 Institute for Health and Nature, Ilisimatusarfik University of Greenland, Nuuk, Greenland

## Abstract

The proceedings of the One Health Gathering in Iqaluit, Nunavut, Canada, centered on the theme of “Elevating Indigenous Voices in One Health Research in the Arctic.” Approximately fifty participants from Greenland, the USA, Canada, and across Nunavut's three regions convened to explore key aspects of research pertinent to this theme. The gathering underscored Indigenous Knowledge and Practices, emphasizing innovative post-secondary education models rooted in Inuit ways of knowing. It also delved into Holistic Worldviews and Health and Wellbeing, spotlighting community country food programs, harvesting practices, and the significance of holistic perspectives in healthcare. Local contexts and community voices were prioritized, with presentations showcasing community-led testing for a parasite in walrus, co-management programs for polar bears, and the vital role of student voices and perspectives. Elder stories and wisdom were shared, adding invaluable insight and depth to discussions. Moreover, the gathering fostered community empowerment and action on One Health research and/or policy, culminating in collaborative recommendations and an art piece aimed at amplifying community engagement and agency in research initiatives. Overall, the event provided a platform for diverse voices to converge, exchange knowledge, and collaborate towards a more inclusive and effective approach to One Health research in the Arctic, reflecting a commitment to Indigenous perspectives, community empowerment, and holistic wellbeing.

## Introduction

Indigenous voices and perspectives on One Health research elevate Indigenous perspectives and holistic models for human-animal-environment interactions, promotes respectful collaboration, and fosters a more comprehensive understanding of health dynamics. Sustainable solutions that benefit both human and animal populations while preserving the environment are key actions moving forward. Elevating Indigenous voices is a crucial step towards achieving equity, justice, and mutual respect in scientific research and decision-making processes throughout the Arctic [1, 2].

The Qaujigiartiit Health Research Centre hosted and organized the One Health Gathering on Arctic Indigenous Peoples Voices and Perspectives February 1–2, 2024, in Iqaluit, Nunavut. The Qaujigiartiit Health Research Centre (QHRC) is an independent, non-profit community research institute that was founded in 2006 by Nunavummiut (Inuit word meaning ‘People of Nunavut’), for Nunavummiut, to answer the health questions of local communities. Their mission is to enable health research to be conducted locally, by northerners, and with communities in a safe, supportive, culturally sensitive, and ethical environment as well as promote the inclusion of Inuit and Western epistemologies and methodologies (ways of knowing and doing) in addressing health concerns, creating healthy environments, and improving the health of Nunavummiut.

The gathering focused on the theme of “Elevating Indigenous Voices in One Health Research in the Arctic” emphasizing the following aspects of research:

- Indigenous Knowledge and Practices;
- Holistic Worldviews and Health and Wellbeing;
- Local Contexts and Community Voices; and
- Community Empowerment and Action on One Health Research and/or Policy.

Approximately fifty (50) participants traveled to Iqaluit from Greenland, the USA, Canada, and all across Nunavut’s three regions. The following text is a summary of the two-day gathering.

## Day 1—February 1, 2024

### Focus: honoring teachings and framing one health

#### Opening and welcoming remarks

The gathering began with a welcome message from Nancy Mike, Research Associate with the Qaujigiartiit Health Research Centre.

The gathering officially opened with the lighting of a *qulliq* by **Elder Annie Petaulassie**. The qulliq is a traditional Inuit oil lamp, typically made from soapstone or other local materials. Annie, a retired teacher and artist from Iqaluit, shared stories from her youth as a young Inuk growing up in Nunavut while bringing light into the room with her process for lighting the *qulliq*. The *qulliq* lighting ceremony is a significant cultural tradition for Inuit, particularly in Canada's Arctic regions, where this gathering is held. It symbolizes warmth, light, community, spiritual connection, and survival for Arctic peoples, serving as the central focus of the ceremony. Annie’s stories and teachings passed down through generations emphasized the significance of the qulliq and its role in Inuit culture and survival. Her insightful teachings to the group were reminders of the respect that humanity must hold for the land and the animals of Nunavut, as well as each other.

She also drew attention to materials for the *qulliq* including the stone for the basin, the plants harvested from the land to make the wick, and the oil which comes from seals. The *qulliq* is a physical, tangible representation of One Health in action, encompassing a vivid animal-environment-human interaction and bringing into a conference setting. The *qulliq* lighting ceremony is ultimately about fostering a sense of community connection, resilience, and strength in the face of challenges, reflecting the values and spirit of Inuit.

Traditional and contemporary Inuit dancing and drumming accompanied the ceremony, adding to the sense of celebration and cultural expression. Annie’s wise words were followed by a moving musical performance by the Inuksuk Drum Dancers, a youth choir and Inuit drum dance group from Inuksuk High School in Iqaluit, Nunavut, Canada.

Overall, the *qulliq* lighting ceremony was a deeply meaningful and symbolic tradition that honors Inuit culture, spirituality, and resilience in the Arctic environment.

#### Keynote Speaker 1—Siila Watt-Cloutier—In attendance by Video Conference Call

*Siila Watt-Cloutier Environmental, cultural, and human rights advocate. She was born in Kuujuuaq, Nunavik, Canada. Siila entered the residential school system when she was 12, which ignited her desire to help others and be of service to her community. She began her career in the Nunavik education system, and eventually became a voice for Inuit rights on the global stage, shining a spotlight on the ramifications of climate change on communities.*

**Siila Watt-Cloutier** brought a wealth of knowledge to the gathering, serving as a fine choice to introduce the two-day gathering. Her deep understanding of the history of the north, paired with the lived experience that her family endured, Siila brought extraordinary knowledge to the gathering.

Watt-Cloutier emphasized the interconnectedness of environmental issues with human rights, particularly for Indigenous peoples of the Arctic whose traditional and contemporary ways of life are deeply intertwined with the natural environment. Watt-Cloutier noted how climate change disproportionately affects Indigenous communities, leading to issues such as food insecurity, loss of cultural identity, and threats to mental and physical health.

*“Culture is medicine. Country Food is medicine. Land is medicine.” – Siila Watt-Cloutier, OneHealth Gathering, Iqaluit, Nunavut, 2024*

Watt-Cloutier also discussed various forms of trauma that have the ability to significantly affect Inuit health including food sovereignty, the RCMP’s slaughtering of sled dogs, addiction, violence, and toxins that travel through the food chain and contaminate breast milk. She emphasized that many of these health traumas emerged in such a short period of time, following the advent of colonialism in the north.

Moreover, she highlighted the importance of recognizing the Arctic as a barometer for global climate change, emphasizing that what happens in the Arctic doesn't stay in the Arctic, but has far-reaching consequences for the entire planet.

Watt-Cloutier advocated for urgent action to address climate change, calling for collaboration, awareness, and policy changes on local and global levels. She also stressed the importance of Indigenous knowledge and leadership in finding sustainable solutions to environmental challenges. Silla ended her presentation by highlighting the significance of the Qaujigiiartiit Health Research Centre in Nunavut for capturing the needs of Inuit and making recommendations based on the research to improve Inuit health.

#### Keynote 2 – Elder dialogue facilitated by Joe Karetak

*Featuring: Joe Karetak, Inuit Qaujigimajatuqangit Researcher for Aqqiumavvik Society, Arviat, Nunavut, Canada. He was the editor for the book Inuit Qaujimajatuqangit What Inuit Have Always Known to Be True and is a former educator. Joe is passionate about making Inuit culture and heritage part of the curriculum for Nunavut Schools.*

**Joe Karetak** shared insight into his experience leading the Elders Advisory Committee. He shared stories about the importance of gathering traditional knowledge from elders which was something he often did during his leadership in the committee.

He was joined by Tunaalaaq Mike Gibbons, Haviq Lisa Gibbons, Paul Sanernut, Lucy Sanernut, and Quluaq Catherine Pilakapsi. The group engaged in a discussion circle on the intersectionality and universality of human, environmental, and animal health.

The panel discussion was lively, filled with personal stories. Each panelist shared stories from their youth and the profound influence of the Elders in their community. They expressed the importance of childhood education. Children gain knowledge by observing and listening to the world around them. In the past, Inuit parents often acted as role models. The group expressed concerns about Inuit family dynamics today, and the diminishing respect towards Elders by youth. In addition, Elders expressed that they felt their peers did not teach as freely as they once did because of the changing nature of relationships. The Elders shared personal accounts of historical trauma inflicted upon their communities, including forced relocations, residential schooling, and cultural suppression. These experiences have had lasting effects on individual and collective well-being, as well as on traditional knowledge systems and cultural practices related to human-animal-environmental health and knowledge.

The Elders discussed how arrival of the European and Canadian explorers, Hudson’s Bay traders, missionaries, and other individuals from the south in the 1920 s marked significant change for Inuit communities. In their discussion, they highlighted how, before this time, there was no formal western-style health system for Inuit, as the Inuit had (and continue to have) a holistic health worldview that enveloped all members of the group in relation to the environment. The discussion emphasized the deep connection that Inuit communities have with the environment and the animals relied on for sustenance, cultural practices, and spiritual beliefs. Elders shared stories illustrating the reciprocal relationship between humans, animals, and the land, highlighting the importance of respecting and stewarding these interconnected ecosystems.

The Elders shared how Inuit way of teaching is very different from the western or eurocentric perspective. There were concerns from the panel about the current education system which is predominantly English-based, rather than Inuit knowledge-based teachings. This resulted in a lack of respect for Elders and their invaluable knowledge. Despite differences in today’s society, respect for each other across ages is necessary., The Elders shared stories of resilience and adaptation within their communities, emphasizing the importance of preserving traditional knowledge and cultural practices in navigating environmental and social changes, even whilst facing significant challenges. They also discussed the role of community-led initiatives and Indigenous-led research in promoting sustainable development and well-being.

Overall, the discussion provided valuable insights into the complex interactions between human societies, animals, and the environment in Nunavut, highlighting the need for holistic approaches to address the interconnected challenges facing Inuit communities.

### Theme 1: Greenlandic perspectives

The afternoon session began with Greenlandic perspectives on the interconnectedness of human, animal, and environmental health. Delegates from Greenland in attendance included Dr. Aviaja Hauptmann, Arnârak Patricia Bloch, Dr. Karsten Rex, Nike, Agathe, Aka, Christina.

**Speaker 1:** Dr. Aviaja Hauptmann.

Title: The SILA Biology Program at Ilisimatusarfik University of Greenland.

**Aviaja Hauptmann,** a Greelandic microbiologist, gave a presentation of the SILA Bachelor of Science program, and how it relates to health. *Sila* is an Inuktitut word which encompasses the weather, the mind, the world order, the spirit, the outside environment, the world, and humanity. The SILA Bachelor of Science program was designed to implement a biology program that would be suited for Greenland, tailored to the Greenlandic way of learning. The three-year program consists of courses grounded in insights from the land, dogs, caribou, and other wildlife. The mission of SILA, a culturally grounded program, is to create a space where Greenlandic youth feel connected to their studies, allowing them to feel more confident, have a greater sense of well-being, and strong mental health through outdoor activities and connection with the land.

**Speaker 2:** Dr. Karsten Rex.

**Dr. Karsten Rex, a practicing family physician** in Nuuk, Greenland, presented on the first-person accounts of bridging western and Kalaallit (Greenlandic) perspectives on health in Greenland. Drawing from his experiences as a Kalaallit health professional, he shared stories and insights from his research which focused on contact tracing, vaccinations, and family guidance on infectious illness in Greenland settlements. As a general practitioner, Karsten described the need to quickly adapt from one patient to another and understand the impacts of the Western lifestyle and how it affects Inuit health.

To conclude the session, other Greenlandic delegates including the Minister of Health, Agathe Fontain, shared their impressions of Nunavut and how conversations with youth and listening to elders during the gathering shaped their perspectives.

### Theme 2: perspectives from: Inuit Nunangat on one health & wildlife

**Speaker 1:** Jason Akearok.

Title: Nanuk Narratives: A co-management collaboration and first-person testimony of Inuit knowledge.

Collaborators: J. Akearok, T. Palliser, J. Snook, D. Borish, A. Caughey, A. Cunsolo, D. Henri.

**Jason Akearok**, a wildlife biologist from Sanirajak/Iqaluit and Executive Director of the Nunavut Wildlife Management Board began the session with his presentation entitled *“Nanuq Narratives”*, which explored knowledge of polar bears through the voices of the people living among them.

Abstract: Polar bears (the Nanuq) are foundational to Inuit health and well-being through livelihoods, food, knowledge, and culture, yet Inuit experience and knowledge related to polar bears is often underrepresented in management decisions and policy making. The goal of this multi-year research project is to gather, preserve, and communicate Inuit Knowledge and experiences with Nanuk in Nunavut, Nunavik, Nunatsiavut, all through community- led, research-based documentary film. Project activities are focused on: 1) orally and visually documenting Inuit knowledge, observations, and relationships with Nanuk; 2) co-creating a video series that highlights the human dimensions of Nanuk relationships; 3) analyzing video interviews to produce qualitative research outputs about Inuit-polar bear connections; and 4) mobilizing research for educational, stewardship, and policy purposes within and outside Inuit communities. This project is an Inuit co-management-led initiative and collaborative effort among the Torngat Wildlife and Plants Co-management Board (Nunatsiavut), Nunavut Wildlife Management Board (Nunavut), Nunavik Marine Region Wildlife Board (Nunavik), as well as multiple Hunter and Trapper Organizations. This project will be a major source of information related to Nanuk for educators, researchers, governments, and public audiences at interjurisdictional levels ranging from the international to the individual.

**Jason Akearok** presented on the importance of polar bears for Inuit communities, including cultural identity, food security, land connection, well-being, and socio-economic benefits. He discussed the importance of hunting and harvesting in his own life, with his wife and children, and importance of taking into consideration the holistic and Inuit-focused approach to co-management of important wildlife populations, such as polar bear. He emphasized the opportunities provided by the use of video as an educational tool and shared the inner-workings of a documentary production currently underway as part of the project.

**Speaker 2:** Dr. Enooyak Sudlovenick.

**Enooyaq Sudlovenick** presented her research conducted in Nunavut and the Northwest Territories, which focused on marine mammals. By using Inuit Knowledge Systems, Ennoyaq discussed the collaboration with One Health as an avenue to explore Inuit health in relation to mammals. She emphasized the Indigenous perspective, which sees humans as part of nature rather than superior to it. Enooyaq would like to see everyone share this story of health that is guided by the Two-Eyed Seeing model which is a way to combine indigenous and western views and ways of knowing.

**Speaker 3:** Jamal Shirley and Sharon Edmunds.

#### A one health approach to trichinellosis prevention – the nunavut trichinella detection program

Abstract: Established in 2017, the Nunavut Trichinella Detection Program (NTDP) is a unique community based zoonotic parasite surveillance program that provides a rapid diagnostic service for Trichinella parasites in harvested wildlife. This disease-causing parasite has historically caused outbreaks of Trichinellosis in communities, impacting well-being. The NTDP was established in response to a long-standing call for testing closer to home, a service based in Nunavut. The program brings together many partners and collaborators across Nunavut, representing Inuit, government and community-based organizations who are united under the umbrella of promoting the continued use of traditional country foods, while protecting human health and building responsive and building accessible laboratory based diagnostic capabilities for zoonotic parasite detection. This presentation will provide an overview of the program’s history, discuss some of the important milestones achieved in its seven years of operation, and share important insights gained from the uniquely collaborative One Health model employed for Trichinellosis prevention in Nunavut.

**Jamal Shirley** and **Sharon Edmunds** on the One Health approach to Trichinella infection prevention. They educated the group on the Trichinella parasite, which attaches itself to omnivores and carnivores, including Orcas, Polar Bear, Beluga, Fox, Wolf and dogs. Their research on trichinella contributes to confirming infections in animal meat and preventing transmission to people. This can better equip communities to prevent mass outbreaks. Jamal and Sharon highlighted the importance of killing the parasite through cooking, which poses challenges for Inuit because much of the diet includes raw meat. The Nunavut Research Institute lab now has the capabilities to test for Trichinella in Arctic mammals harvested in Nunavut.

## Day 2—February 2, 2024

### Theme 3: from personal stories to collaboration to action

The second day of the gathering began with a teaching from Elder Mike Gibbons (Tunaalaak) on how to maintain well-being in an Inuit lifestyle. He acknowledged the One Health gathering as a great example. He highlighted that the gathering provides balance, wellness, and knowledge-sharing across colonial borders, recognizing that all of Inuit Nunangat were gathered in the room. Elder Mike remembers the pre-colonized Inuit way of life. It was full of health—country food was healthy, people were happy. Today, Inuit are constantly navigating a new way of living, facing new challenges while dealing with their own trauma. This is all in pursuit for true happiness and to live a rich life. Elder Mike concluded with a prayer.

Enooyaq recapped the previous day's teachings before introducing the speakers of the day. The first part of the morning was dedicated to *Storytelling and Knowledge Exchange: Narratives, Stories, Art, Film.*

**Speaker 1:** Nancy Mike.

**Nancy Mike** presented *Inuit Qaujimajatuqangit and One Health: Exploring Multi-Faceted Perspectives on Inuit Health Systems.*

This presentation delved into the intricacies of Inuit health systems and perspectives within the framework of the One Health paradigm. Rooted in rich cultural Inuit knowledge, the discussion highlights the holistic worldview, which encapsulates a comprehensive understanding of human-animal-environmental interdependencies. At the heart of this discussion lies Inuit Qaujimajatuqangit (IQ) and the recognition of the profound intersectionality between human, animal, and environmental health. Inuit, as stewards of the Arctic environment, maintain a unique symbiotic relationship with the land, wildlife, and communities.

Nancy chose not to use PowerPoint, instead she shared stories that she had written for children based on her childhood experiences. “Hunting with Dad” shared the tale of an eight-year-old girl in Pang who has to choose between the western way of life and the Inuit way of life.

This presentation explored the multi-faceted nature of the Inuit Qaujimajatuqangit worldview, and how it operates as a system. We examine how Inuit knowledge, community-based approaches, and cultural practices are integrated to address health concerns holistically, emphasizing prevention, resilience, and sustainability.

Through stories and collaborative art, this presentation offered insights into the strengths and challenges of helping people understand the complexities of the Inuit systems worldview, which is broader and richer than typical academic approaches to One Health. Nancy presented a graphic on the health system model for Inuit, soon to be published in the Inuit Studies Journal [3]. The graphic shows a model for health from the Inuit worldview which is holistic in that it includes the land, kinship connection, language, connection to animals, and acknowledging the realms beyond human understanding. By fostering a deeper understanding of the Inuit Qaujimajatuqangit paradigm, we aim to cultivate a more inclusive and effective approach to health that transcends cultural boundaries and advances the wellbeing of all interconnected beings. This model can exist in harmony with the existing western model when it is acknowledged and uplifted.

**Speaker 2:** Karen Aglukark.

**Karen Aglukark** spoke on *Healing and Transformation: An Inuk Student’s Journey from the Western Classroom Back to the Community.*

Abstract: This researcher’s project aims to understand the relationships and community supports that contribute to, and are transformed through, the process of healing from childhood adversity. Guided by Inuit research methodologies, this study serves as a foundational step in a proposed wider community-based participatory research project by first consulting with an advisory group to determine the suitability and scope of the research question and objectives. Challenges experienced by the researcher in defining this project are discussed: Post-secondary mental health education programs predominantly align with Western paradigms, which poses several challenges for students aspiring to serve Inuit; As well, pathways for careers in mental health services are largely focused on Western approaches to health and service-delivery; It is therefore challenging for students to meaningfully prepare for research and work conducted from within Indigenous paradigms and methodologies. While a growing body of research highlights the importance of integrating relational epistemology and holistic approaches to health in Inuit communities, there are few supports that guide Inuit students on how to integrate these approaches in their academic and professional careers. This presentation outlines the researcher’s journey as they shifted their career from Western psychological approaches to holistic wellness. This discussion also sheds light on the challenges faced when integrating Indigenous knowledge as the foundation of master’s level research projects. Ensuring that students are exposed to and educated on community-specific research methods and ways of knowing will be integral to successfully translating Indigenous and community knowledge into relevant health and healing services in our communities.

Through personal stories, Karen shared stories from her past and how those experiences led her to being a successful woman in the field of health and research. She used her adversity as motivation, drawing strength, and using it as fuel to help others in her community. She shared her story as an example to other Inuit graduate students and early career scholars on the challenges and strengths that come with being in a research field, particularly related to community health and wellbeing.

**Speaker 3:** Rhoda Katsak and Damian Enoogoo.

**Project:** Inuksiutit: Inuit Food Sovereignty in Nunavut. Mittimatalik, Nunavut. The Inuksiut project is is funded through the Canada-Inuit Nunangat-United Kingdom (CINUK) Arctic Research Program in collaboration with York University (Canada) and the University of Aberdeen (Scotland).

**Rhoda Katsak** is an Inuit Knowledge Holder from Mittimatalik, Nunavut, and Co-Investigator with the Inuksiutit research project.

**Damian Enoogoo** is a 19-year-old film-maker from Mittimatalik, Nunavut.

Abstract: The Inuksiutit: Inuit Food Sovereignty in Nunavut project (IFSNu) aims to promote the intergenerational transmission of country food knowledge and foster community health and wellbeing through capacity-building in younger generations of Inuit. This session will share community-produced short films on the topics of Inuit food, animals, and environmental change, showcasing the power of community action on One Health research which reflects Inuit holistic views of health. The films include demonstrations on how to harvest, process, and prepare different traditional foods, as well as interviews and conversations with Elders and youth about environmental change and solutions for supporting food sovereignty. This session featured films produced by the Dana Katsak, the Katsak family, and Damian Enoogoo in Mittimatalik, and Neevee Jaw, Martha Jaw, and Aaron Pudlat in Kinngait.

**Damian Enoogoo** began the presentation by sharing the story of his youth and how that shaped his future as a photographer and videographer. Damian concluded that his work with the Inuksiutit Food Sovereignty project was a highlight of his year and has made him think about continuing his education and training in the film area of study. Videos by Damian and Dana Katsak: www.youtube.com/@ifsnuinuitfoods5435Rhoda Katsak discussed the centrality of Inuit food, country food, in both her own life and in the context of community health, well-being, food security and food sovereignty. She elaborated on intergenerational film production to support Inuit food knowledge, sharing reflections on Inuit youth today and discussing the importance of youth learning about Inuit culture, food and the way of life. Rhoda’s passion for country food and knowledge around harvesting and preparing country food is a bridge to connect with the younger generation. Rhoda worked with her granddaughter to create a video to help teach youth, and expressed that media was the best way to captivate the younger generation.

*“Seal meat and fat keep us warm. We need the heat from seal meat and fat to keep our bodies warm. Our bodies crave seal meat and fat. We crave the vitamins. We need this… people cannot afford the meat sold in the stores…”*

### Theme 4: country food and food sovereignty

**Speakers:** Igah Sanguya, Martha Jaw, Dr. Amy Caughey.

**Project:** Niqivut Silalu Asijjipalliajuq (NSAP): Our food & climate change: Country food for health and well-being in Nunavut. NSAP is funded by the Canadian Institutes of Health Research in collaboration with the University of Alberta School of Public Health (Canada). Nunavut partners include Nunavut Tunngavik Incorporated, Government of Nunavut, and QHRC.

**Abstract:** Country food is critical to health and well-being for Inuit communities. Inuit women and their families hold unique and important knowledge of Inuit food systems, including knowledge and skills related to country food preparation and preservation which are essential for the continued use of country food. The “Niqivut Silalu Asijjipalliajuq (NSAP): Our food & climate change” research program privileges community-held knowledge in supporting food sovereignty, food safety, healthy eating and country food as medicine. More broadly, our research program centers on holistic community and environmental health, bringing together animal-human–environment knowledges to support Inuit health and well-being in Nunavut. This presentation will discuss how our work was led by Inuit women in Nunavut, and share findings of our research to date.

**Igah Sanguya** is an Inuit Knowledge Holder from Clyde River, Nunavut, and a Research Associate with the University of Alberta School of Public Health. Igah worked for many years in the health system in Nunavut, as a community health representative. Along with her family, Igah spends much of her time on the land and at her cabins, hunting and fishing. Igah described that country food is at the core of food sovereignty in Nunavut, emphasizing the importance of country food in her life, and how many Inuit can relate to this. Igah spoke to the importance of Inuit knowledge sharing related to country food and food systems, for overall community health and well-being. She wanted the group to understand the importance of offering country food to Elders, in particular at times of illness, as they often crave it and have less access to it than in the past.

*“Our country food is our medicine.”*

*-Igah Sanguya*

**Martha Jaw** is an Inuit Knowledge Holder, Community Health Representative, mother, grandmother and great-grandmother from Kinngait, Nunavut. Martha is a community collaborator on the Inuksiutit Food Sovereignty project, and on the NSAP country food and climate change project. Martha shared stories from her home community and knowledge about preparing difference types of harvested foods, including important and exciting foods such as a women’s whale tail feast, and clams from the stomach of a walrus. Martha spoke to photos of country food that she and others in the team have prepared, preserved, and documented in the name of sharing this knowledge with others in the community. Martha and Igah screened a short video they produced entitled *NSAP: Our Food & Climate Change, (*https://vimeo.com/905620394/7d8d434956?share=copy*)* describing Inuit womens’ perspective on the importance of country food and climate change in Nunavut.

**Amy Caughey** is a non-Indigenous nutritionist and public health researcher living in Iqaluit. She is a Research Associate and Adjunct Professor with the University of Alberta, and a Research Associate at the Nunavut Research Institute in Iqaluit. Amy provided a summary of the NSAP program, and highlighted recent work from the research team, including engagement with the National Inuit Strategy on Research (NISR) in food sovereignty research in Nunavut (Caughey et. al, 2022), and the meaning of country food for Inuit women (Caughey et al., 2024). She emphasized that the work of the NSAP research team highlights the significance of country food for overall nutrient intake and food security in Nunavut and supports country food as a critical component of physical and mental health Inuit in Nunavut.

### Theme 5: building partnerships and visioning for the future

In the afternoon, the gathering participants formed two groups.

- The first group engaged in a **“Creative Collection”** which entailed arts-based discussions. The discussion was facilitated by Nancy Mike and Dr. Gwen Healey Akearok.
- The second group formed a **“Discussion Circle”** and was facilitated by Arnârak Patricia Bloch, Dr. Aviaja Lyberth Hauptmann, Dr. Karsten Rex, Dr. Christina Larsen.

The following instructions were given:

**Table:** 

Group 1	Group 2
• Draw a piece of art to represent what One Health means to you. • The group came up with a drawing made of bright colours featuring a rainbow representing hope. The piece emphasized the importance of family, land, animals and youth. It carried the message that humanity is just one facet of the world, everything is important and interconnected. • The outcome of this exercise was that art is such an important tool for expression and connection, especially when not everyone can verbally express their thoughts.	• In this discussion circle, participants were invited to reflect on and share perspectives on the following: • What are community questions/concerns/research for OneHealth? • What is needed to support Indigenous perspectives, what is the message up to international groups, such as the Arctic Council/Sustainable Development Working Group? • Should this gathering be repeated?

Overall, the participants expressed a desire for more opportunities to gather in this way, it left them feeling excited and motivated. They wanted to inquire about the creation of gathering spaces within their communities.

*“When we gather, culture is the medicine.” – participant response*

*“A safe and stable home is the foundation of health. Having a safe space allows the welcoming of more gatherings to be organized.” – participant response*

The participants'concerns included how difficult it is to try to reach the younger generation.

Priorities included encouraging them how to express their individuality, and how to persevere and not give up. Advice from participants for the younger generation included, but was not limited to:

- Do not hide your Inuit identity.
- The past cannot be changed.
- Communication is key.
- Be proud of being Inuk/Indigenous.
- Embrace the future.
- Culture is different for everyone, embrace your unique way of living, and educate others.
- Arctic people are resilient (referring to TB, suicide, etc.).

Numerous difficult stories shared during the gathering but hearing about solutions from across the Circumpolar countries helped to balance the trauma and challenges.

“While listening is good, acting is better”—participant

Participants were happy to hear of all the resources that exist and wanted those working in One Health to actively seek collaboration to make meaningful change.

### Call to action

The discussion concluded with a call to action, urging greater recognition of Indigenous knowledge systems, meaningful engagement with Arctic Indigenous communities, and collaborative efforts to address the root causes of environmental degradation and health disparities. Finding a way to meaningfully collaborate between Indigenous and non-Indigenous worldviews was seen as critical and necessary in the advancement of One Health perspectives. Elders emphasized the importance of empowering future generations to continue the legacy of stewardship and resilience in the face of ongoing challenges.

### Closing circle and farewell

The gathering ended with a closing circle bringing together the whole group for final comments. The gathering sparked learning opportunities, meaningful discussions, forward thinking ideas, networking opportunities and relationship building opportunities. The participants were impressed to learn about the diverse projects across Inuit Nunangat and around the Arctic. In particular, participants noted the benefits derived from close collaborations between Nunavut and Greenland, and the potential for more essential knowledge exchange and actions between these regions. One Greenlandic participant noted, *“[Nunavut] is the only place we feel at home away from home.”*

The young leaders and the Elders were acknowledged, and served as a reminder that collaboration across generations is essential for progress in Arctic communities to move forward in a good way.

Indigenous voices and perspectives on One Health research elevates Indigenous perspectives and holistic models for human-animal-environment interactions, promotes respectful collaboration, and fosters a more comprehensive understanding of health dynamics. Sustainable solutions that benefit both human and animal populations while preserving the environment are key actions moving forward. Elevating Indigenous voices is a crucial step towards achieving equity, justice, and mutual respect in scientific research and decision-making processes.

## Data Availability

Not applicable.
